# Relapsing–remitting multiple sclerosis patients exhibit differential natural killer functional subpopulations

**DOI:** 10.1007/s13760-024-02488-y

**Published:** 2024-03-05

**Authors:** Inês Rodrigues Barreto, Andreia Monteiro, Artur Paiva, Ana Mafalda Fonseca

**Affiliations:** 1 CICS-UBI – Health Sciences Research Centre, University of Beira Interior, Av. Infante D. Henrique, 6200-506 Covilhã, Portugal; 2 Clinical Pathology Service, Centro Hospitalar Universitário da Cova da Beira (CHUCB), Covilhã, Portugal; 3 Cytometry Operational Management Unit, Clinical Pathology Service, CHUC, Coimbra, Portugal; 4 iCBR – Coimbra Institute for Clinical and Biomedical Research, University of Coimbra, Coimbra, Portugal; 5 ESTESC-Coimbra Health School, Coimbra, Portugal

**Keywords:** Relapsing–remitting multiple sclerosis, Natural killer cells, IFN-γ, TNF-α, IFN-β therapy

## Abstract

**Background:**

Multiple sclerosis (MS) is a chronic inflammatory disease of the central nervous system (CNS) and has been known as T-cell mediated. However, the contribution of multiple cell types, notably natural killer (NK) cells, has also been reported.

**Aim:**

To quantify circulating total NK cells and its subpopulations, CD56 dim and bright, and to characterize the functional phenotype and IFN-γ and TNF-α production in relapsing–remitting patients treated with IFN-β and in apparently healthy controls.

**Results:**

CD56^bright^ NK cells were found to be the least represented subpopulation. In relapse patients, the frequencies of IFN-γ-producing NK cells and their subpopulations were significantly decreased. In remission patients, CD56^dim^ NK cells expressed high levels of HLA-DR and CD54.

**Conclusion:**

These results suggest that remission RRMS patients, although in an inactive stage of MS, present circulating NK cells with an activation phenotype, supporting the idea that NK cells may be relevant mediators in the MS pathophysiology.

## Introduction

Multiple sclerosis (MS) is a chronic inflammatory disease that leads to the demyelination and profound destruction of the central nervous system (CNS). Relapsing–remitting is the most frequently diagnosed phenotype and is defined as an initial episode of neurological incapacity, followed by a period of clinical recovery. As the disease progresses, a state of neurological disability is expected to settle [1].

MS is characterized as an autoimmune disease which was traditionally considered to be mainly mediated by autoreactive T cells that, unexpectedly, began to infiltrate in the blood–brain barrier (BBB) [2]. However, the role of other immune cells [3], such as B cells [4], in MS is now recognized, due to their presence in demyelinating plaques and in inflammatory infiltrates, but also because of new therapeutical approaches. The introduction of an anti-CD25 antibody that blocks the α-component of the IL-2 receptor (IL-2R) [5] and inhibits T-cell activation proved critical in recognizing the role of natural killer (NK) cells in MS, as these cells expanded with this therapy [6]. A dysfunction in NK cell-mediated regulatory features was also described in untreated MS, suggesting a contribution to MS pathogenesis [7]. An expansion of NK cells in MS patients treated with interferon (IFN)-β has also been reported [8, 9].

NK cells, phenotypically defined as lacking CD3, and differentially expressing CD16 and CD56, are essential innate immune system agents, known as cytotoxic cells, lysing target cells without specific immunization [10]. Two subpopulations can be defined based on the relative expression of CD56, bright and dim, which influences the function played by each subpopulation. CD56^bright^ NK cells show a high expression of IL-2R, are recognized as immunoregulatory, and display a superior cytokines production, namely IFN-γ and TNF-α [11, 12]. CD56^dim^ NK cells are more abundant within peripheral blood and have a much greater cytotoxicity, with more perforin, granzyme, and cytolytic granules than CD56^bright^ NK cells [13, 14].

In MS and in experimental autoimmune encephalomyelitis (EAE), the animal model for neuroinflammation, the role played by NK cells has been contradictory, with both protective and destructive effects being mentioned [15–17]. Recent research has also highlighted the role played by several cell surface markers, like the activation marker HLA-DR [18], the integrin CD11c [19] and the adhesion molecule CD54 [20], in the pathogenesis of MS and EAE.

Therefore, our work aimed to quantify total NK cells, CD56^bright^ and CD56^dim^ NK cell subsets, as well as their production of IFN-γ and TNF-α, in RRMS patients treated with IFN-β and compare them with apparently healthy controls. Some functional properties of CD56^dim^ NK cells were also analyzed to better characterize this subpopulation.

## Materials and methods

### Patients and healthy controls

For this study, 37 patients, diagnosed with RRMS according to the McDonald criteria 2010 [21], were enrolled and divided considering the phase of the disease: 29 patients in remission and 8 patients in relapse.

The inclusion criterion was IFN-β therapy, whereas the exclusion criteria were the use of corticosteroid treatment or other MS therapies, active infection, local or systemic illness affecting the immune system (such as chronic inflammation or other autoimmune disorders) and pregnancy. A relapse was defined as an acute inflammatory demyelinating episode in the CNS lasting at least 24 h, without fever or infection, and verified by neurological findings. Disability was assessed with the Expanded Disability Status Scale (EDSS), ranging from 0 to 10, with higher scores indicating more disability.

Twenty apparently healthy age- and gender-matched volunteers were recruited as healthy controls (HC).

All patients and volunteers signed an informed consent, and the study was approved by the ethics committee of Centro Hospitalar Universitário Cova da Beira. Table 1 represents the demographic and clinic characteristics of RRMS patients and HC.

**Table 1:** Demographic and clinical characteristics of RRMS patients and HC. Data are expressed as mean ± standard deviation (SD) or median (inter quartile range, IQR)

	Relapse patients (*n* = 8)	Remission patients (*n* = 29)	HC (*n* = 20)
Age (mean ± SD, years)	41 ± 15	44.2 ± 11.2	48 ± 9
Male (%)	37.5	10.3	20
Female (%)	62.5	89.7	80
Leukocytes (mean ± SD × 10^9^ L^−1^)	8.4 ± 4.9	6.6 ± 2.0	7.1 ± 2.0
EDSS-score (median (IQR))	2.3 (4)	1.5 (1.5)	NA
Age at onset of disease (mean ± SD)	36.3 ± 14	32.2 ± 9.7	NA
Disease duration (median (IQR))	3.5 (11)	8.5 (10)	NA
*Treatment*			
IFN-beta 1a SI 44 µg 3 × week (*n*)	1	3	NA
Length of treatment (mean ± SD, years)	3	5.7 ± 4.0	NA
IFN-beta 1a IM 30 µg 1 × week (*n*)	3	14	NA
Length of treatment (mean ± SD, years)	9.0 ± 4.2	6.4 ± 2.3	NA
IFN-beta 1b IM 250 µg every other day (*n*)	4	10	NA
Length of treatment (mean ± SD, years)	5.7 ± 1.2	4.8	NA
IFN-beta 1b IM 250 µg every other day (*n*)	0	2	NA
Length of treatment (mean ± SD, years)	0	4.0 ± 1.4	NA

*NA* not applicable, *EDSS* Expanded Disability Status Scale, *SI* subcutaneous injection, *IM* intramuscular injection, *SD* standard deviation

### Frequency of peripheral blood NK cell subsets

Approximately, 200 μL of peripheral blood (PB) sample was collected in K3-EDTA and stained with a combination of mouse anti-human antibodies, detailed in Table 2 (tubes 1 and 2), and incubated for 15 min at room temperature in the dark. A lysis technique was then carried out with FACS Lysing Solution (BD Biosciences), and a washing step was performed. The cells were subsequently resuspended in 500 μL of PBS (PBS; Gibco, Paisley, Scotland) and acquired using a FACSCantoTMII (BD) flow cytometer and FACSDiva software (version 6.1.2: BD). The samples were acquired with established standardized instrument settings recommended by the Euroflow consortium [22]. The number of events collected was usually more than 0.5 × 10^6^. Infinicyt (version 1.8) software (Cytognos SL, Salamanca, Spain) was used for data analysis.

**Table 2:** Panel of monoclonal antibody reagents (with clones and commercial source) used for the immunophenotypic characterization of peripheral NK cell subsets

Fluorochrome								
**Tube**	**V500**	**PacB**	**FITC**	**PE**	**PC5**	**PC7**	**APC**	**APCH7**
**1**	**CD3** SP34-2 BD Pharmingen	**CD8** RPA-T8 BD	**CD27** M-T271 BD Pharmingen	**CCR5** G155-178 BD Pharmingen	**CD56** N901 Beckman–Coulter	**CD45RO** UCHL1 Beckman–Coulter	**CD45RA** HI100 BD	**CD4** SK3 BD
**Tube**	**PacO**	**PacB**	**FITC**	**PE**	**PercP**	**PC7**	**APC**	**APCH7**
**2**	**CD45** HI30 Invitrogen	**HLA-DR** L243 BioLegend	**CD16** 3G8 BD Pharmingen	**CD54** LB-2 BD	**CD11c** N418 BioLegend	**CD33** D3HL60.251 Beckman–Coulter	**CD123** 9F5 BD	**CD14** MϕP9 BD

Immunofluorescence staining after in vitro activation								
**Tube**	**V500**	**PacB**	**FITC**		**PC5**			
**3**	**CD3** SP34-2 BD Pharmingen	**CD8** B9.11 Beckman Coulter	**cyTNFα** AAb11 BD Pharmingen		**CD56** N901 Beckman Coulter			
**Tube**	**V500**	**PacB**	**FITC**		**PC5**			
**4**	**CD3** SP34-2 BD Pharmingen	**CD8** B9.11 Beckman Coulter	**cyIFNγ** 4S.B3 BD Pharmingen		**CD56** N901 Beckman Coulter			

The analysis of these cells was integrated in a panel where other subpopulations were also analysed.

BD Pharmingen (San Diego, CA, USA), Biolegend (San Diego, CA, USA), BD (Becton Dickinson Biosciences, San Jose, CA, USA), Beckman Coulter (Miami, FL, USA), Invitrogen (Life Technologies, Carlsbad, CA, USA); APC, Allophycocyanin; APCH7, allophycocyanin-hilite7; FITC, Fluorescein isothiocyanate; PacB, Pacific blue; PE, Phycoerythrin; PC5, Phycoerythrin–cyanine 5; PC7, Phycoerythrin–cyanine 7; Perc-P, Peridinin chlorophyll protein

To quantify the NK cell subsets, first, total lymphocyte population was identified based on forward (FSC) and side scatter (SSC) properties. Within this cell population, total NK cells were identified as CD3^−^ and according to their CD56 and CD16 surface density expression, respectively as CD56^dim^ CD16^+^ (CD3^−^CD56^low^CD16^+^) and CD56^bright^ CD16^−^ (CD3^−^CD56^high^CD16^−^) [23]. CD8^+^ NK cells were identified among CD3^−^CD56^+^CD16^+^ cell population based on CD8 expression (tube 1). To investigate the functional properties of CD56^dim^ NK cells, the expression of HLA-DR, CD54 and CD11c were analysed.

**Fig. 1:**
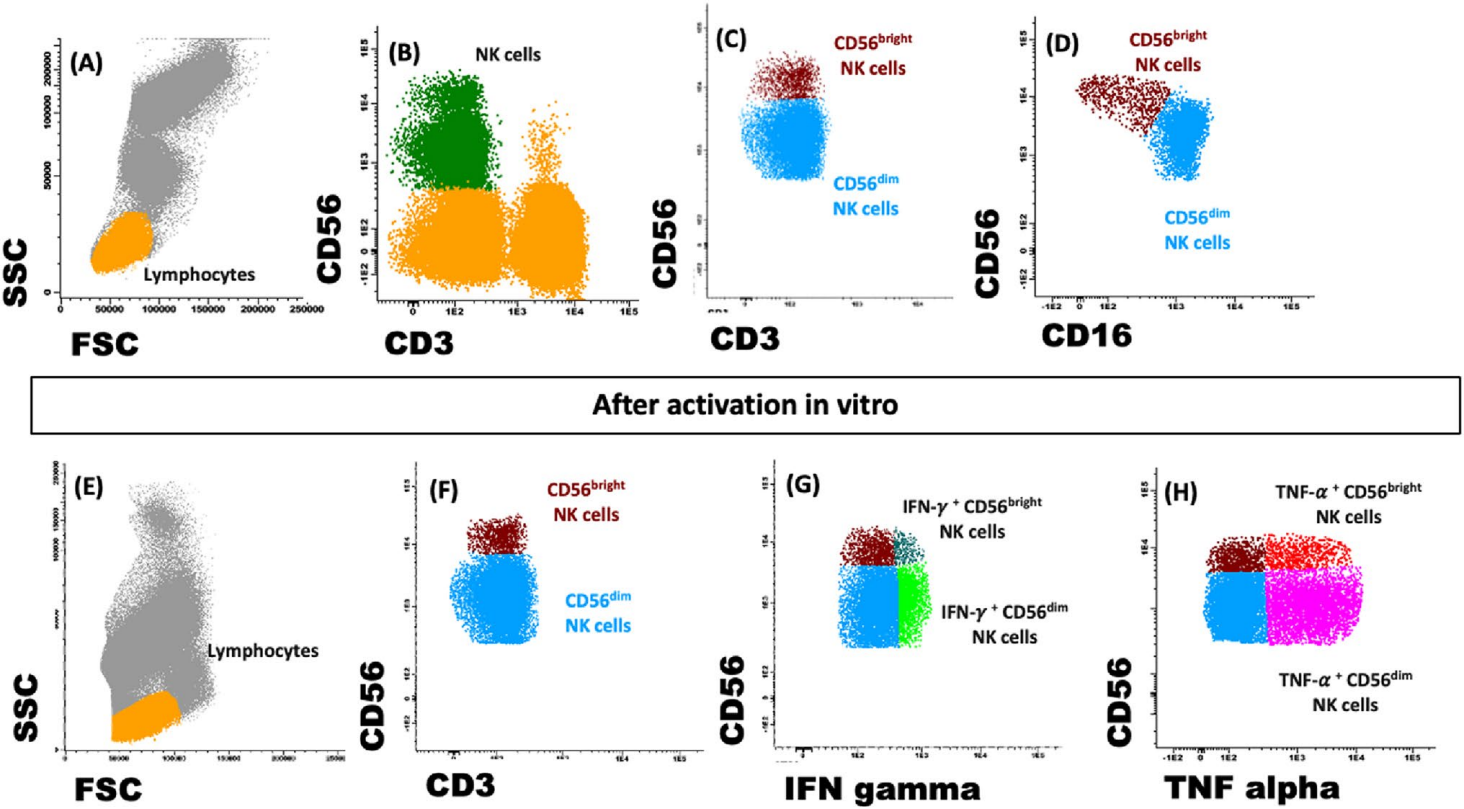
Flow cytometry analysis strategy. Within the total lymphocyte population (gating based on SSC and FSC, **A**), total NK cells population can be identified, according to the CD3 negativity and CD16 and CD56 surface density expression, and subsequently, the subpopulations CD56^dim^ (CD3^−^CD56^low^CD16^+^) and CD56^bright^ (CD3^−^CD56^high^CD16^−^) (**B**, **C**, **D**). After activation in vitro, the lymphocytes were identified (**E**), according to the expression of CD3 and CD56 the NK subsets were identified (**F**), IFN-γ (**G**)and TNF-α (**H**)-producing NK cell subsets were also identified

Absolute counts were calculated using a dual platform methodology (flow cytometer and haematological cell analyser). Results illustrate the percentage of positive cells within each subset. Figure 1 exemplifies a strategy of flow cytometry analysis to identify NK cell subpopulations.

### In vitro* stimulation with PMA/ionomycin, in the presence of brefeldin A*

For each PB sample, aliquots of 500 μL of total blood were diluted 1/1 (vol/vol) in RPMI-1640 medium (Gibco; Paisley, Scotland, UK), supplemented with 2 mM l-glutamine and incubated at 37 °C in a sterile environment with a 5% CO_2_ humid atmosphere for 4 h in the presence of 10 μg/ml of brefeldin A (Golgi plug_Sigma, Saint Louis, MO, USA), 50 ng/ml of phorbol 12-myristate 13-acetate (PMA; Sigma, Saint Louis, MO, USA) and 1 μg/ml of ionomycin (Boehringer Mannheim, Germany).

### ***Intracellular cytokine production by CD56***^***bright***^*** and CD56***^***dim***^*** NK cells***

After the activation period, samples were aliquoted in different tubes (200 μL of PB sample/tube). The samples were initially stained for the surface antigens (Table 2, tubes 3 and 4) incubated for 15 min in the dark at room temperature (RT), washed with 2 mL of PBS and centrifuged at 2000 rpm for 5 min. To analyze the intracellular expression of IFN-γ and TNF-α in NK cells, an intracytoplasmic permeabilization and staining protocol was followed according to the manufacturer’s instructions, Fix&Perm (Caltag, Hamburg, Germany). The cell pellets were resuspended in 100 μL of Reagent A (Fixation Medium), incubated for 15 min in the dark at RT and centrifuged at 2000 rpm for 5 min. Next, 100 μL of Reagent B (permeabilization medium) was added, followed by monoclonal antibodies anti-IFN-γ and TNF-α (10 μL of each) for an incubation period of 15 min. Then, samples were washed with 2 mL of PBS and centrifuged at 2000 rpm for 5 min. After this step, the cell pellets were resuspended in 500 μL of PBS and immediately acquired. CD56dim NK cells were identified based on the expression of CD16 and SSC, selecting the CD16 + population, and next, for analysis of each marker, either CD54 or HLA-DR or CD11c/SSC dot plots were used. Results illustrate the percentage of NK cell subsets producing cytokines and the mean fluorescence intensity (MFI) of IFN-γ and TNF-α.

### Statistical analysis

The statistical evaluation was performed using a non-parametric test, the Kruskal–Wallis test, and post hoc pairwise comparisons with Dunn’s test with Bonferroni corrections for multiple testing. Results were expressed as median (inter quartile range, IQR). In what regards demographic and clinical characteristics of the volunteers, data are presented as mean ± standard deviation (SD) and median (inter quartile range, IQR) was used for description of normally and non-normally distributed data, respectively. All statistical analyses were performed using SPSS software program (version 28.0). *p* values <0.05 were considered statistically significant.

## Results

### Frequency and absolute numbers of peripheral blood NK cell subsets

Among the three groups, the least represented subpopulation was the CD56^bright^ NK cells. A significant decrease in the absolute numbers of total NK and CD56^dim^ NK cells in remission RRMS patients was observed when compared with HC (*p* = 0.047 and *p* = 0.043, respectively). Concerning the frequencies of NK cells and its subpopulations, no significant differences were observed (Table 3).

**Table 3:** Frequency and absolute value of NK, CD56^dim^, CD56^bright^, CD8^+^, CD56^dim^ CD8^+^ and CD56^bright^ CD8^+^ NK cells in RRMS patients and HC

	Relapse patients (*n* = 8)		Remission patients (*n* = 29)		HC (*n* = 20)	
	%	cells/μL	%	cells/μL	%	cells/μL
NK	6.9 (7.3)	111.5 (108.7)	4.8 (6.2)	**72.9 (63.5)**^a^	4.9 (3.3)	120.2 (72.4)
CD56^dim^ CD16^+^	91.7 (13.2)	86.1 (89.8)	89.8 (12.4)	65.3 (51.2)^a^	91.8 (9.3)	101.1 (48.7)
CD56^bright^ CD16^−^	7.8 (8.3)	8.9 (7)	11.1 (12.3)	8.2 (8.7)	9.2 (8.7)	8.6 (9.2)
CD8^+^ NK	34.3 (17.3)	37.9 (45.5)	**12.2 (9.9)**^a,b^	**6.6 (4.5)**^a,b^	22.7 (19)	36.7 (39.8)
CD56^dim^ CD8^+^	96.3 (4.7)	36.8 (45.6)	94.4 (9.7)	**5.1 (4)**^a,b^	95.7 (7.5)	32.8 (36.5)
CD56^bright^ CD8^+^	3.7 (4.7)	1.7 (3.8)	8 (10.9)	**0.4 (0.5)**^a,b^	5.5 (7.7)	1.8 (1.3)

Results are expressed as median (IQR); *p* values were determined by Kruskal–Wallis test and post-hoc pairwise comparisons with Dunn’s test with Bonferroni corrections for multiple testing; statistically significant differences (*p* < 0.05) were observed between ^a^Remission patients vs. HC, ^b^Remission patients vs. Relapse patients, identified in bold

A significant decrease was also found in the frequency and absolute number of CD8^+^ NK cells (identified as CD3^−^CD56^+^CD8^+^ cells, within total NK cell subset) in remission RRMS patients, when compared with relapse RRMS patients (*p* = 0.001 and *p* = 0.009, respectively) and HC (*p* = 0.014 and *p* < 0.001, respectively). The same pattern was also observed regarding the absolute values of CD56^dim^ CD8^+^ and CD56^bright^ CD8^+^ NK cells in remission RRMS patients, as compared to relapse RRMS patients (*p* = 0.008 and* p* = 0.009, respectively) and HC (*p* < 0.001 for both variables) (Table 3).

### Frequency of IFN-γ and TNF-α-producing NK cells subpopulations

**Fig. 2:**
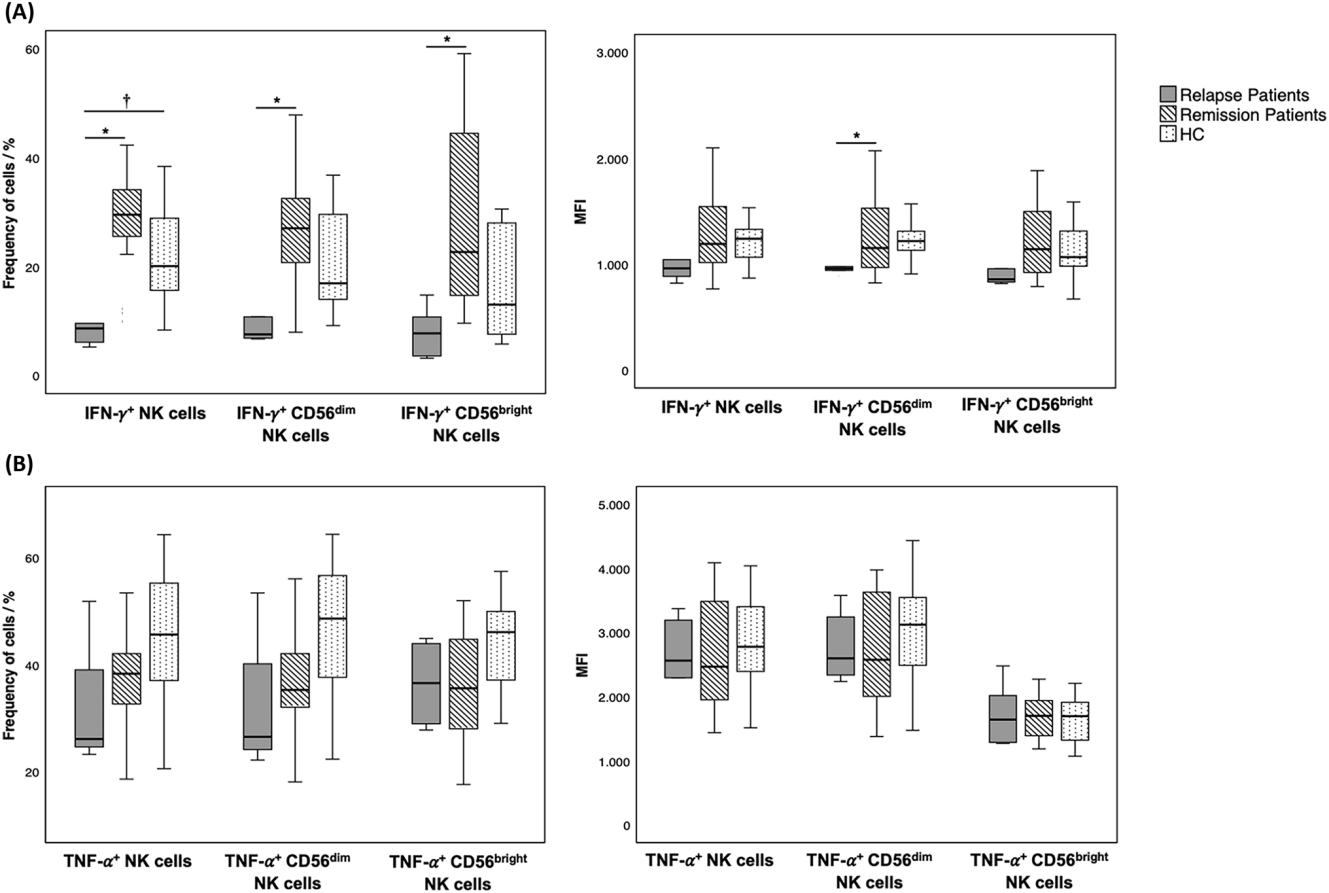
Frequency of **A** IFN-γ and **B** TNF-α-producing NK cells subpopulations. Boxplot represents the median ± IQR; *p* values were determined by Kruskal–Wallis test and post hoc pairwise comparisons with Dunn’s test with Bonferroni corrections for multiple testing; statistically significant differences (*p* < 0.05) observed between *relapse patients vs. remission patients and ^†^relapse patients vs. HC

After in vitro stimulation with PMA and ionomycin, the frequency of NK cell subsets producing IFN-γ and TNF-α was measured. Although our main focus was to study the more frequent circulating subpopulation (CD56^dim^ NK cells) with a recognized immune surveillance function, we also analysed the CD56^bright^ NK cells, which are known to be high cytokine producers. A significant decrease was found regarding the frequencies of total NK cells producing IFN-γ in relapse RRMS patients, when compared with remission RRMS patients and HC (*p* = 0.002 and *p* = 0.039, respectively). Moreover, a significant decrease was observed in the MIF of IFN-γ-producing CD56^dim^ NK cells in relapse RRMS patients, as compared to remission RRMS patients (*p* = 0.048). The same was observed for the frequencies of CD56^dim^ and CD56^bright^ NK cells producing IFN-γ in relapse RRMS patients when compared with remission RRMS patients (*p* = 0.021 and *p* = 0.002, respectively). No significant differences were observed regarding the frequencies of TNF-α-producing NK cell subsets (Fig. 2). Concerning the cytokine-producing CD8^+^ NK cells, we observed that the frequency of IFN-γ and TNF-α-producing was scarce (data not shown).

### ***Phenotypic characterization of CD56***^***dim***^*** NK cells***

We decided to better characterize CD56^dim^ NK cells, since these sub-population was the most represented among RRMS patients and HC, when compared to CD56^bright^ NK cells. Thus, we analysed the expression of the activation marker HLA-DR, the integrin CD11c and the adhesion molecule CD54. Regarding the frequency of HLA-DR^+^ CD56^dim^ NK cells, a significant increase was observed in remission RRMS patients as compared to relapse RRMS patients (*p* < 0.001) and HC (*p* < 0.001). Considering the frequency of CD11c^+^ CD56^dim^ NK cells, significant differences were found among the three groups. Additionally, a significant decrease in the MFI of CD11c in CD56^dim^ NK cells was observed in both relapse and remission RRMS patients when compared to HC (*p* = 0.038 and* p* < 0.001, respectively). Lastly, a significant increase in the frequency of CD54^+^ CD56^dim^ NK cells was observed in remission RRMS patients when compared with HC (*p* = 0.001) (Table 4).

**Table 4:** Phenotypic characteristics of CD56^dim^ NK cells

	Relapse patients (*n* = 8)		Remission patients (*n* = 29)		HC (*n* = 20)	
	%	MFI	%	MFI	%	MFI
HLA-DR^+^ CD56^dim^ NK cells	2.4 (2.5)	733.5 (526.6)	**8.1 (7.4)**^a,b^	996 (628.4)	2.8 (3)	878.2 (579.4)
CD11c^+^ CD56^dim^ NK cells	**81.4 (16.6)**^a^	**2134.7 (1244.8)**^a^	**65.4 (18.1)**^a,b^	**1913 (1944.4)**^a^	49.8 (11.2)	922.1 (827.6)
CD54^+^ CD56^dim^ NK cells	3.4 (1.8)	603.7 (277.7)	**5.5 (4.7)**^a^	562.1 (345)	2.8 (1.8)	622.2 (139.6)

Results are expressed as median (IQR); *p* values were determined by Kruskal–Wallis test and post hoc pairwise comparisons with Dunn’s test with Bonferroni corrections for multiple testing; statistically significant differences (*p* < 0.05) were observed between ^a^remission patients or relapse patients vs. HC, ^b^remission patients vs. relapse patients, identified in bold

## Discussion

The immunopathogenesis of MS is still unknown, but there is increasing evidence that NK cells may be relevant to the disease development and progression [24]. In this study, the results suggest that both the frequency and absolute number of circulating NK cell subsets are altered in RRMS patients. First, it was found that the CD56^bright^ NK cell subset was the least represented among the studied groups. As an immunoregulatory subpopulation playing a protective role in MS [6, 25] and given the fact that, after a year [8] or two [9] of IFN-β treatment it appears to expand, a higher circulating frequency of this subpopulation was expected. As the patients analysed here have been submitted to this therapy for 3 year or more, perhaps a long-term use of IFN-β may mask these changes.

In addition, a significant decrease was observed in the frequency of CD8^+^ NK cells in remission RRMS patients. Although it was not part of the initial aim, this finding seems relevant since a decrease in a CD8^low^CD56^+^CD3^−^CD4^−^ cell subpopulation was reported in non-treated RRMS patients. Furthermore, the same subset was found to be increased after anti-CD25 antibody treatment [6], and associated with brain inflammation reduction [26], suggesting these have a regulatory function. Moreover, a population of ‘NK8^+^ cells’ (CD3^−^CD56^+^CD8^+^) has also been described and correlated with promising clinical outcomes in MS [27]. Given this, we believe that this subpopulation needs to be further characterized phenotypically and functionally, to ensure it is the same as those previously described.

After in vitro stimulation with PMA and ionomycin, similar frequencies of CD56^bright^ and CD56^dim^ NK cells producing IFN-γ were observed overall among the three groups. It is accepted that CD56^bright^ NK cells are the main IFN-γ production subset, and especially after being stimulated [28]. The limited number of relapse patients may explain the lack of statistical difference obtained, at least in part. Also, those authors propose that the CD56^bright^ subset starts cytokine production at later intervals, so that may also impact the observed results. Between groups, when considering pairwise comparison, we found a significant decrease in the frequency of IFN-γ-producing NK cell subpopulations in the relapse group when compared with the remission RRMS patients and HC. This may result from the exiting of these cells from peripheral blood contributing to the relapse and progression of MS, and/or because of the effects of the regulatory circuits of the therapy.

Lastly, from the phenotypic characterization of CD56^dim^ NK cells, we observed that remission patients were found to have increased frequencies of cells expressing HLA-DR and CD54 (when compared with relapse patients), while the contrary was observed for the frequency of CD11c-expressing NK CD56^dim^ cells. The expression of both HLA-DR and CD54 on NK cells seems to be relevant in the context of autoimmune diseases, like MS and EAE, respectively, contributing to the production of IFN-γ [29] and promoting T cell adhesion and leucocyte migration through the BBB [20]. Likewise, and although being known as an activation marker for monocytes/macrophages [30], the expression of CD11c on NK cells in RRMS may be associated with the temporal activity of the disease [19]. The results suggest that the remission RRMS patients, supposedly in an inactive disease state, seem to have circulating CD56dim cells with an activation phenotype, characterized by the expression levels of HLA-DR. Interestingly, higher frequencies of CD54-expressing NK cells were found among remission RRMS patients, meaning that perhaps NK cells are capable of migrating to the inflammation site. To achieve more significant results, the expression of a broader group of molecules, to functionally characterize NK cells and its subpopulations, should be investigated.

This study has limitations regarding the flow cytometry methodology, since we did not use antibodies to CD3, CD16, and CD56 in all tubes of the monoclonal antibodies panel. Moreover, FMO (fluorescence minus one) controls were not incorporated in the study, as well as CD14 and CD19/CD20 markers, which would exclude without any doubt CD14 monocytes (although they have distinct FSC/SSC characteristics comparing to lymphocytes) and CD19 B cells from the analysis. Another limitation is concerned with the number of patients included in the MS groups. Consequently, the expression levels of these patients vary considerably, leading to variable values between groups. Yet, since most publications study relapse and remission patients globally and do not acknowledge the differences that seem to exist between them, it can be considered a worthy point of the research [31]. It should be noted that, regarding the demographic and clinic characteristics of RRMS patients and HC, the sample does not show statistically significant differences in age, gender, and EDDS (Expanded Disability Status Scale).

Moreover, we believe that ideally, we should follow the same patient in both phases of RRMS and compare each immunological profile along the time and include a group of untreated RRMS. Furthermore, it would have been relevant to analyse cerebrospinal fluid (CSF) samples as well. However, taking all this into account, we believe that this study can give important clues for the importance of monitoring NK cells in RRMS patients treated with IFN-β.

## Conclusion

There is evidence on the essential role NK cells play in regulating the mechanisms involved in the development and progression of MS; however, their specific role is still unclear. The results seem to confirm that NK cells play an active role in the different phases of RRMS. In future, to generate more consistent findings, it would have been necessary, for example, to have a similar number of participants across all groups and to analyse CSF samples as well. A broad understanding of how their mechanisms of action influence the immune responses may prove important to pave the way for the development of novel medicines targeted at improving the lives of affected individuals.

## Data Availability

The data that support the findings of this study are available from the corresponding author, [Fonseca AM], upon reasonable request.
